# Interactive effects of personal resources and job characteristics on mental health: a population-based panel study

**DOI:** 10.1007/s00420-020-01555-0

**Published:** 2020-06-06

**Authors:** Anja Limmer, Astrid Schütz

**Affiliations:** 1 Department of Psychology, University of Bamberg, Bamberg, Germany; 2 Chair of Personality Psychology and Psychological Assessment, University of Bamberg, Markusplatz 3, 96047 Bamberg, Germany

**Keywords:** Mental health, Self-esteem, Locus of control, Job demands, Job resources, Interactive effects

## Abstract

**Purpose:**

We examined 10 job characteristics in a large population-based sample and tested for positive and negative effects on mental health. In addition, we tested for possible effects on mental health from interactions with locus of control and self-esteem.

**Methods:**

The sample comprised longitudinal data on 2353 male and 1960 female employees from the German socio-economic panel collected between 2010 and 2012. Mental health was assessed with the mental component summary score derived from the short-form 12 health survey. We computed hierarchical regression analyses while controlling for potential confounds and baseline mental health. Interaction effects were specified with post hoc simple slope analyses.

**Results:**

Time pressure, interruptions, job insecurity, and conflicts were negative predictors of mental health in all models. The personal resource of self-esteem was a positive predictor. Moreover, there were interactions: opportunities for promotion were beneficial only for employees with medium or high levels of self-esteem, whereas the contrary was true for employees with very low self-esteem. Working on weekends was negatively related to mental health for people with moderate to low internal control but not for people with high internal control.

**Conclusions:**

The findings suggest that there are job demands that are related to poor mental health regardless of personal resources. These aspects are important to consider in workplace risk assessment. By contrast, with other job characteristics (e.g., opportunities for promotion, weekend work), the effects vary between individuals.

**Electronic supplementary material:**

The online version of this article (10.1007/s00420-020-01555-0) contains supplementary material, which is available to authorized users.

## Introduction

Because of its fundamental importance for an individual’s quality of life, mental health is often considered the key target of occupational health policies (Badura 2017). Furthermore, mental illness is among the leading causes of why people may be unfit to work (e.g., Whiteford et al. 2013), and stress or strain may even be passed from leaders to their employees (Köppe et al. 2018).

In searching for ways to protect employees’ mental health, job characteristics have become widely accepted as relevant factors in the fields of occupational health and work design (Harvey et al. 2017; Parker et al. 2017). Meta-analyses have identified relations between certain job characteristics and depression (Theorell et al. 2015; Kim and Knesebeck 2016), burnout (Aronsson et al. 2017; Lee and Ashforth 1996), and common mental disorders in general (Stansfeld and Candy 2006). It has been shown that many job characteristics can be considered to be job demands (e.g., long working hours, job insecurity, or workplace conflicts) because they require extra effort and are a threat to mental health (Demerouti et al. 2001). By contrast, other job characteristics such as job autonomy and social support can be considered to be job resources (Luchman and González-Morales 2013) because they help people meet job requirements and thus reduce job strain and associated health risks and can even stimulate personal development (Bakker and Demerouti 2017; Demerouti et al. 2001).

For yet other job characteristics, however, the evidence is less clear: There are some (mostly correlational) findings on relations between shift work and health problems (Lee et al. 2017). But other studies have found that the impact of shift work depends on the specific type of shift work (Zhao et al. 2019), as well as on contextual and individual factors (Tahghighi et al. 2017). Similarly, job characteristics such as time pressure, interruptions, or poor chances of being promoted have been found to be jointly associated with an increased risk of mental problems (Stansfeld and Candy 2006; Siegrist et al. 2009), but most studies have not provided information about their incremental impact (Luchman and González-Morales 2013). In sum, the impact of many job characteristics is still not clear. In particular, their correlations with mental health might be overrated if effects of personality are neglected (see Alarcon 2011).

Employee personality has been shown to be consistently associated with burnout (Alarcon et al. 2009) and the ability to cope with professional demands (Gottschling et al. 2016) in cross-sectional studies. Direct effects have been found for relatively stable personality traits (e.g., emotional stability; Østby et al. 2018), as well as for more malleable personal resources. Personal resources were defined by van den Heuvel et al. (2010) as “lower-order, cognitive-affective aspects of personality” (p 129), which constitute developable positive beliefs about oneself and the environment. A substantial body of evidence has shown that personal resources such as self-efficacy, self-esteem, optimism, and locus of control are important predictors of mental health (Boudrias et al. 2014; Mäkikangas et al. 2004) and that they lower the risk of burnout (Alarcon et al. 2009).

In our reading of the literature, two very broad resources stand out with respect to their potential impact: self-esteem and internal locus of control. Global self-esteem refers to a person’s overall self-evaluation of his or her worth (Rosenberg 1965) and has been reported to be one of the best predictors of mental health in the work context (Kalimo et al. 2002; Mäkikangas et al. 2004). The concept of internal locus of control describes the extent to which individuals believe they can determine events in their own lives (Rotter 1966) and has demonstrated empirical relations with a broad range of work outcomes, particularly mental well-being (Alarcon et al. 2009; Ng et al. 2006).

In the present study, we examined seven job demands as well as three job resources and tested their direct effects on mental health as well as their interactions with personal resources (i.e., locus of control and self-esteem) in a large sample of German employees in various occupations. We expected that long working hours, shift work, weekend work, time pressure, frequent interruptions, job uncertainty, and conflicts at work would have a negative impact on mental health, but aspects of social support or autonomy, and promotion opportunities would have a positive impact. We further expected positive associations between personal resources and mental health as well as interactions with job characteristics. Because confidence in one’s own abilities and a feeling of mastery helps people cope with demanding circumstances and make use of opportunities (Hobfoll 1989), we expected self-esteem and internal locus of control to act as buffers against the negative effects of job demands and to boost the positive effects of job resources.

## Methods

The sample was derived from three waves of the German socio-economic panel study (GSOEP 2017), a longitudinal representative survey of German employees. In this panel, a biannually mental health score has been generated since 2002, and supplementary questions about work characteristics and personal resources have been included since 1999 at varying intervals.

Accordingly, balanced panel data, collected in 2010 (time 1), 2011 (time 2), and 2012 (time 3) were used to test the hypotheses described above. Person- (time 1) and job-related (time 2) predictors as well as baseline mental health (time 1) were assessed prior to the outcome measure of mental health at time 3. Our analysis was limited to adults with full-time or regular part-time employment (excluding part-time workers with unpredictable schedules, apprentices, and people in sheltered employment) who had not yet reached the legal retirement age of 65 years. To minimize interfering or reversed influences, respondents who had changed jobs at time 2 or time 3 were excluded. The resulting sample of *N* = 4313 participants included 45.4% women and 54.6% men.

## Measures

### Job characteristics

We measured the actual number of hours worked per week as a continuous variable assessed with one GSOEP item [“how many hours do your actual working hours consist of, including possible overtime?” (Wagner and Schupp 2012)]. In the GSOEP, shift work was assessed with a question relating to different work schedules (“do you sometimes have to work evenings or nights?”) with five response categories ranging from 1 (no) to 5 (yes, every day). We categorized shift work as 1 if the answer was “yes, once a week (changing shifts)” for either evenings or nights and as 0 for any other response. Working on weekends was assessed as the sum of the hours given by participants in response to questions about how many hours they spend on their job on a typical saturday or sunday. Time pressure, interruptions, job insecurity, and the chance of being promoted were measured with the corresponding single items from a short version of the ERI questionnaire, which had been validated previously (Siegrist et al. 2009). For each aspect, the respondents first agreed or disagreed, and subsequently, those who agreed were asked to rate the extent to which they felt burdened on a 4-point scale. We followed the procedure proposed by Siegrist et al. (2009) and recoded both answers to a 5-point Likert scale (whereby the chance of being promoted was reverse-keyed).

The GSOEP generates a sociological measure of autonomy in occupational activity developed by Hoffmeyer-Zlotnik (2003) by using detailed information on a person’s occupational position. A score ranging from 1 (low autonomy in occupational activity) to 5 (high autonomy in occupational activity) is coded on the basis of dimensions such as company size, employment sector, or vocational training [see SOEP Group (2012) for details]. Unlike other sociological measures associated with job status, autonomy in occupational activity focuses on the differences in autonomy and authority within occupations (Kröger 2017). This approach is also conceptually different from self-report measures, as it reflects the formal level of autonomy at the workplace, which can be seen as a predictor of actual job autonomy (Kröger 2017). Evidence for the relations between the approaches can be found in a factor analysis of autonomy in occupational activity and self-rated questions about variety in job tasks and working method autonomy by Fahr (2011), who found one underlying factor on which the three measures loaded almost equally. Because there was no explicit measure of social support in the workplace, we generated a variable for the number of trusted or career-supporting individuals in the workplace: In the GSOEP, respondents were asked to name up to five individuals they confide in and five individuals who have supported the advancement of their careers. We combined support from supervisors and coworkers into one dichotomous variable with a value of 1 if colleagues and supervisors were named by the respondents and 0 if not. Conflicts at work were assessed on the basis of a question about people with whom respondents had arguments or conflicts. If colleagues or supervisors were mentioned, the answer was coded 1 for conflicts at work; if not, it was coded 0.

### Personal resources

Locus of control was assessed with a scale developed by Nolte et al. (1997). The items (e.g., “how my life goes depends on me”) were rated on a 7-point scale ranging from 1 (not at all) to 7 (absolutely). In accordance with Specht et al.’s (2013) specifications, we aggregated the scores into a scale with higher values indicating internal locus of control and a reported Cronbach’s alpha coefficient of 0.70 for this wave (Richter et al. 2013). Self-esteem was measured with the single item, “i have a positive attitude toward myself,” which has been shown to be reliable and valid in adult samples (Robins et al. 2001). Respondents were asked to indicate their agreement with this statement on a scale ranging from 1 (does not apply to me at all) to 7 (applies to me perfectly).

### Mental health

Mental health was assessed with the mental components summary (MCS), which was derived from the short-form 12 health survey (SF-12; Andersen et al. 2007). The MCS aggregates six items on vitality, social functioning, emotional functioning, and overall mental health (i.e., “during the last 4 weeks, how often did you feel calm and relaxed?”). This composite score of mental health is standardized and so ranges from 0 to 100 with higher values indicating better mental health, and the mean and standard deviation of every survey wave set to 50 and 10, respectively. It has been found to be reliable and valid (*α* = 0.78; Andersen et al. 2007).

### Control variables

Previous work has examined the association between mental health and demographic factors. Whereas a meta-analysis found that older employees do not suffer a decline in mental health (Ng and Feldman 2013), later studies proposed that age can moderate the associations between job characteristics and occupational stress and strain (Zacher and Schmitt 2016). For gender, there is evidence that the impact of job demands on mental health differs between women and men (Stansfeld and Candy 2006). In addition, studies have found that education is associated with job characteristics (Lunau et al. 2015), mental health (Bjelland et al. 2008), or both (Milner et al. 2018). Thus, we used these characteristics as control variables. Age was measured as a continuous variable. *Gender* was categorized as 1 for men and 0 for women. Education level was based on the International standard classification of education (ISCED) and combined into five groups (ranging from 0 = primary and lower secondary education to 4 = master, doctoral, or equivalent level). Furthermore, to control for potential differences due to the hierarchical position, leadership was assessed with the item, “in your position at work, do you supervise others? In other words, do people work under your direction?” and coded 1 (yes) or 0 (no).

## Statistics

Descriptive analyses and hierarchical linear regression analyses were computed using STATA/SE 14.2. Because of minor imperfections in the data distributions (e.g., skewness of age), we used the robust Huber-White standard error sandwich estimator, which provides accurate inferences in large samples (Lin 2013). Potential confounds as well as the baseline measure of mental health were controlled for in step 1. Job demands and job resources were entered in step 2, followed by personal resources (step 3) and all two-way interactions between job characteristics and personal resources (step 4). When the interaction between person- and job-related characteristics emerged as significant, post hoc simple slope analyses were computed and plotted at one standard deviation above and below the mean of personal resources (Dearing and Hamilton 2006). Preacher, Curran, and Bauer’s (2006) calculator was used to estimate the upper and lower bounds of their regions of significance on the basis of the Johnson-Neyman technique (Hayes and Rockwood 2017). Prior to the analyses, all metric and ordinal predictors, except for education level and weekend work hours, were mean-centered. Preliminary analyses of missing data showed an arbitrary pattern of <5% missing values for all variables, justifying listwise deletion (Baguley and Andrews 2016), especially in the context of multiple regression (Graham 2012).

## Results

Descriptive statistics can be found in Table 1 for all variables. Table 2 presents the results of the hierarchical regression models. After adjusting for baseline mental health and potential confounds (step 1, *R*^*2*^ = 0.29), the set of 10 job characteristics explained an additional 2.1% of the variance in mental health (∆*R*^*2*^ = 0.02, *p* < 0.001). Weekend work, time pressure, frequent interruptions, job insecurity, and conflicts at work were negatively related to mental health. Autonomy in occupational activity and the chances of being promoted were positive predictors of mental health, whereas hours worked per week, shift work, as well as trust and career-support at work showed no significant effects.

**Table 1:** Descriptive statistics of the study sample (*N* = 4313)

Variables	Range	*N*	%	Average	SD
Mental health 2012 (MCS)	13–70			49.8	9.2
Mental health 2010 (MCS)	8–79			50.3	9.1
Age (years)	20–62			45.1	9.6
Gender					
Men		2353	54.6		
Women		1960	45.4		
Education					
Primary/lower secondary		233	5.4		
Upper secondary		2050	47.5		
Post-secondary		540	12.5		
Bachelor or equivalent		1051	24.4		
Master/doctoral or equivalent		439	10.2		
Leadership					
Yes		1531	35.5		
No		2782	64.5		
Shift work					
Yes		521	12.1		
No		3792	87.9		
Working hours (per week)	3–80			40.9	10.7
Weekend work (hours)	0–48			3.0	5.1
No work on weekends		2623	60.8		
Between 1 and 48 h per weekend		1690	39.2		
Time pressure	1–5			2.5	1.3
Interruptions	1–5			2.4	1.3
Job insecurity	1–5			1.3	0.8
Conflicts at work					
Yes		788	18.3		
No		3525	81.7		
Trusted or career-supporting individuals					
Yes		1338	31.0		
No		2975	69.0		
Autonomy in occupational activity	1–5			3.0	1.0
Chances of promotion	1–5			3.9	1.1
Self-esteem	1–7			5.6	1.2
Locus of control	1–7			4.9	0.9

**Table 2:** Multiple regression analyses predicting mental health (*N* = 4313)

	Step 1		Step 2		Step 3		Step 4		
	*b*	*β*	*b*	*β*	*b*	*β*	*b*	*β*	*p*
Baseline mental health	0.53***	4.86***	0.49***	4.45***	0.44***	4.03***	0.44***	3.98***	0.000
Age (years)	0.02	0.23	0.02	0.22	0.02	0.23	0.03*	0.26*	0.032
Male (0 = no, 1 = yes)	0.50*		0.50		0.52		0.53*		0.046
Education level									
Upper secondary	1.12		1.06		1.13		1.13		0.059
Post-secondary	0.80		0.75		0.76		0.83		0.220
Bachelor or equivalent	1.32*		1.13		1.16		1.19		0.069
Master/doctoral or equivalent	1.07		0.77		0.77		0.79		0.288
Leadership (0 = no, 1 = yes)	0.39		0.45		0.33		0.34		0.231
Working hours			0.00	0.05	− 0.00	− 0.01	− 0.00	− 0.04	0.792
Shift work (0 = no, 1 = yes)			0.45		0.38		0.40		0.277
Working on weekends (0 = no, 1 = yes)			− 0.75**		− 0.78**		− 0.75**		0.002
Time pressure			− 0.51***	− 0.65***	− 0.50***	− 0.64***	− 0.47***	− 0.60***	0.000
Interruptions			− 0.32**	− 0.40**	− 0.31**	− 0.39**	− 0.32**	− 0.40**	0.004
Job insecurity			− 0.42**	− 0.35**	− 0.38*	− 0.32*	− 0.33*	− 0.28*	0.032
Conflicts at work (0 = no, 1 = yes)			− 0.95**		− 0.85**		− 0.78*		0.015
Trusted or career-supporting individuals (0 = no, 1 = yes)			− 0.12		− 0.17		− 0.18		0.489
Autonomy in occupational activity			0.31*	0.33*	0.29	0.30	0.27	0.28	0.087
Chances of promotion			0.41***	0.47***	0.36**	0.41**	0.35**	0.40**	0.002
Self-esteem					0.82***	0.96***	0.79***	0.93***	0.000
Internal locus of control (LoC)					0.28	0.26	− 0.07	− 0.06	0.740
Self-esteem × promotion							0.29**	0.39**	0.003
Internal LoC × weekend work							0.97**		0.002
Total *R*^*2*^	0.29		0.31		0.32		0.33		
Adjusted *R*^*2*^	0.29		0.31		0.32		0.32		
∆*R*^*2*^	0.29***		0.02***		0.01***		0.01**		

Unstandardized coefficients (*b*) for all predictors and standardized beta-coefficients (ß) for non-categorial variables. Whereas all possible two-way interactions between personal resources and job characteristics were added in step 4, the table presents only those with significant effects (*p < 0.05, **p < 0.01, ***p < 0.001)

Adding personal resources in step 3 accounted for an additional 1.1% of the variance (∆*R*^*2*^, *p* < 0.001) in mental health. Self-esteem showed a significant positive main effect, whereas internal locus of control did not. The effects of all but one job characteristic (autonomy in occupational activity) remained significant (*p* < 0.05).

When adding the two-way interactions between personal resources and job characteristics, the overall model (step 4) was still significant, *F* (40, 4272) = 46.03, *p* < 0.001, and an additional 0.7% of the variance in mental health (∆*R*^*2*^, *p* < 0.01) was explained, for a total adjusted *R*^*2*^ value of 32.4%.

**Fig. 1:**
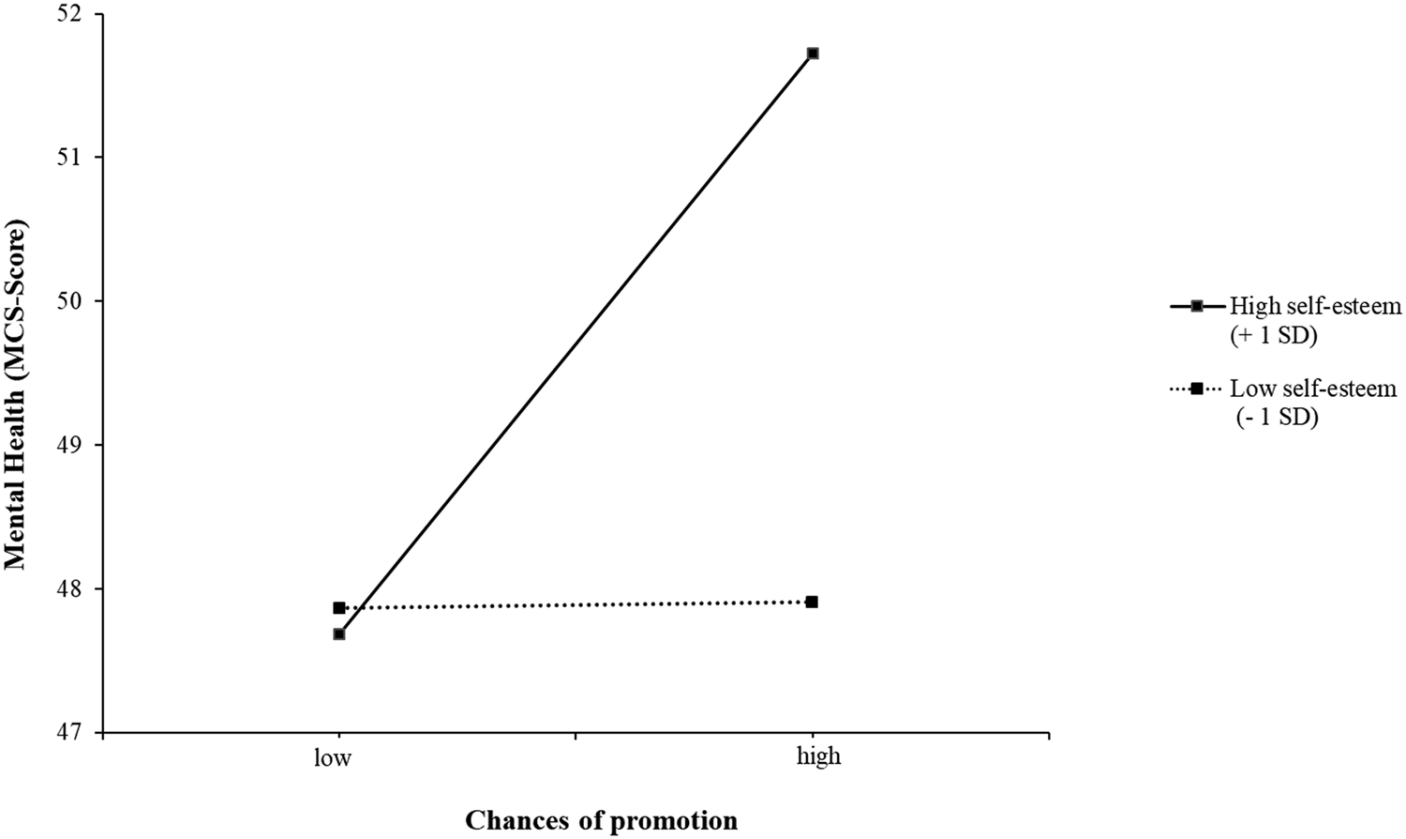
Interaction between self-esteem and chances of promotion in predicting the mental health of German employees: post hoc simple slope analysis (*N* = 4313)

In addition, the effects of job characteristics and personal resources were qualified by statistically significant interactions between self-esteem and the chance of being promoted as well as the interaction between internal locus of control and number of hours worked on weekends. As can be seen in Fig. 1, the simple slope tests revealed that mental health was highest when both the chance of being promoted and self-esteem were high. Further, the region of significance for self-esteem was outside the lower bound of − 3.88 and the upper bound of − 0.40, whereas the centered self-esteem values ranged from − 4.63 to + 1.37. Taken together, it can be concluded that the effect of the chance of being promoted on mental health was negative for people with very low (below − 3.88) values of self-esteem and had an increasingly positive effect at medium and high (above − 0.40) levels of self-esteem.

**Fig. 2:**
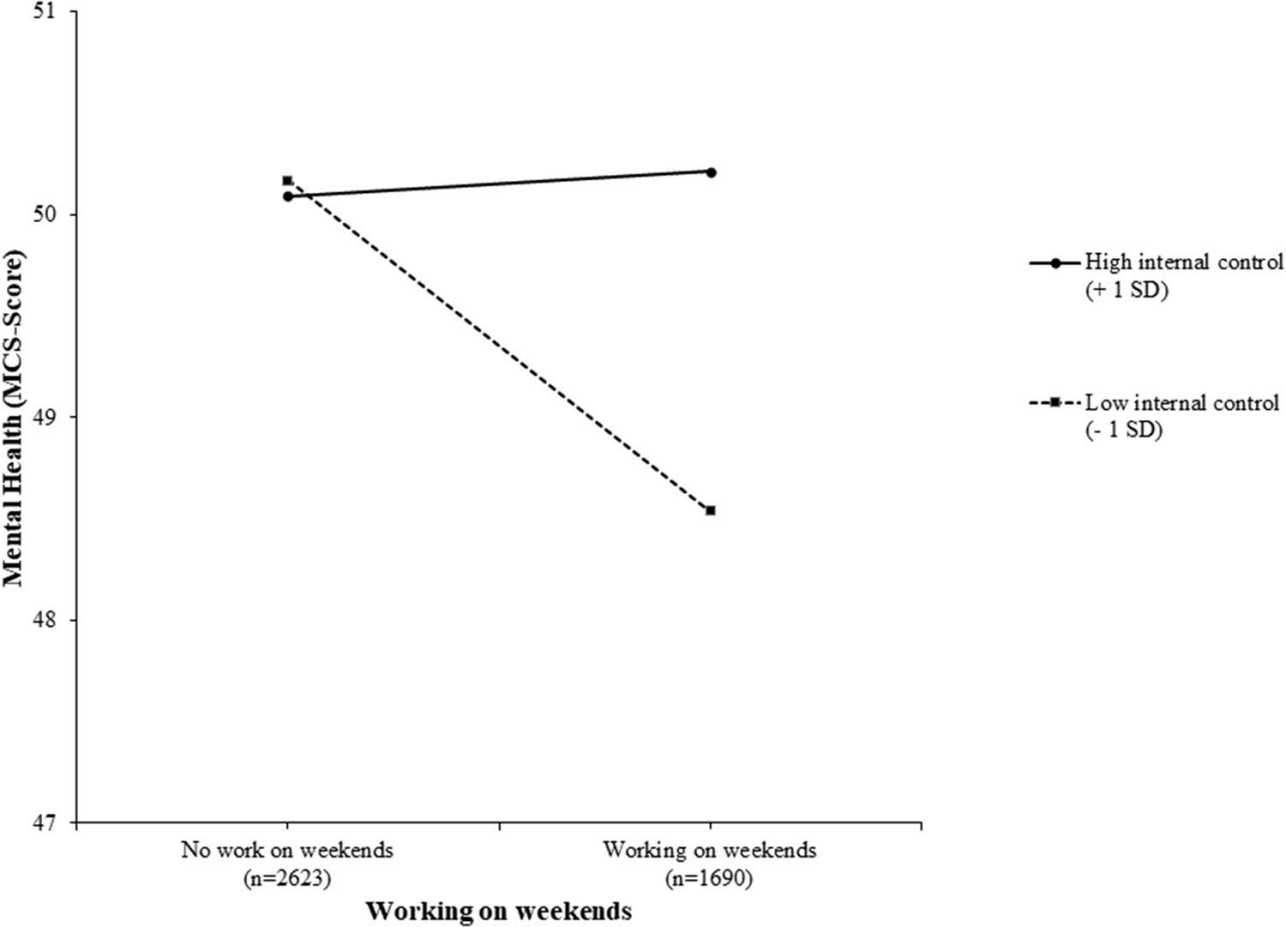
Interaction between internal locus of control and weekend work in predicting the mental health of German employees: post hoc simple slope analysis (*N* = 4313)

Simple slopes for the interaction between locus of control and working on weekends (see Fig. 2) were negative for low (*b* = − 1.63, *t* = − 4.20, *p* < 0.001) and medium (*b* = − 0.75, *t* = − 3.04, *p* < 0.01) levels of internal control but positive for high (*b* = 0.12, *t* = 0.33, *p* = 0.74) levels. More precisely, the region of significance for internal locus of control was limited to values lower than 0.26 and higher than 2.22. Given that the centered values of internal locus of control ranged from − 3.29 to 2.13, these results indicate that mental health was negatively associated with working on the weekends for respondents with moderate to low internal control. However, for respondents with above-average levels of internal control, working on weekends had no significant effect on mental health.

## Discussion

The goal of this research was to contribute to the existing knowledge about predictors of well-being in work situations by investigating how various job characteristics and personal resources interact and affect mental health. To our knowledge, this is the first study to use a population-representative sample and longitudinal data to study the impact of global ratings as well as more objectively measured indicator-based measures of job characteristics and their interactions with personal resources on mental health across occupations.

Across several models, we found evidence for the associations of high time pressure, frequent interruptions, high job insecurity, and conflicts at work with impaired health and for the role of a good chance of being promoted as a job resource. In line with the job demands-resources model (Demerouti et al. 2001) and considering the cross-sectoral and cross-occupational nature of our data, these findings suggest that global ratings of job characteristics predict mental health regardless of industry sector, profession, hierarchy, or personality. Perceived time pressure, interruptions, job insecurity, and chances of promotion were assessed as part of a scale based on the effort–reward imbalance model (Siegrist 1996) and resulted in health effects that were in the same direction as those previously observed for the compound measures: high time pressure and frequent interruptions are two out of three aspects that represent high effort, whereas job insecurity and poor job promotion constitute aspects of low reward. High effort and low reward both predict reduced employee health (e.g., Harvey et al. 2017). In the light of recent calls by these authors for research on clusters of work stress rather than single models, our results support the notion that the specific job characteristics we examined have effects on health that are similar to those from the joint ERI compounds and that they even show incremental effects beyond additional concepts of work environment. Nevertheless, future research is needed to confirm the validity of these single item measures.

Overall, the effect of a single job demand or resource was relatively small—less than 1 point of the mental health score (MCS) when all other predictors were controlled for—which is not surprising given the multicausal nature of health (Zapf et al. 1996) and considering that even relatively small effect sizes can result in nontrivial growth in risk for employees in the most risky or demanding situations, as argued by Ford et al. (2014). Furthermore, in the present study, we did not include reciprocal effects between mental health and job characteristics—an approach that has recently been shown to increase explained variance (Lesener et al. 2019).

The more objectively measured and indicator-based job characteristics—i.e., working hours, shift work, autonomy (based on occupational position) and social support operationalized by listing individuals who are perceived as trusted or supportive of one’s career—did not (consistently) impact mental health. In some cases, such as for working hours, the lack of effect on mental health could also be due to underlying curvilinear relations (Warr 1987). However, preliminary explorations of their relations as well as post hoc inspections of the augmented partial residual plot did not indicate a clear departure from linearity. Another explanation for the lack of an effect could be the use of indicator-based measures as opposed to more global ratings. For example, for working hours, it might not be the number of hours actually worked that are associated with mental health outcomes but rather a mismatch between the numbers of actual and desired work hours (De Moortel et al. 2018). Similarly, the measure of autonomy in occupational activity was based on objective aspects of an employee’s occupational position and might therefore not be comparable to self-reported feelings of autonomy. In line with the concept of tied autonomy (Väänänen and Toivanen 2018) and recent attempts to disentangle different dimensions of work autonomy (e.g., Spiegelaere et al. 2016) a refined assessment may provide clearer evidence as well as starting points for preventive health interventions. The same reasoning applies to our finding that the number of trusted or career-supporting individuals at work was not a positive predictor of mental health. In contrast to typical measures of social support, which were based on overall ratings, we assessed support from only one domain (work) and on the basis of whether participants had listed colleagues or supervisors when recalling individuals who supported their career and in whom they confided. Although such support is certainly important, apparently the measure differs from previously studied aspects of social support such as a friendly atmosphere in everyday conversation or overall supervisor support. More systematic exploration and more valid measures are needed to better understand the forms and functions of support and to identify specific predictors of mental health. Finally, the finding that shift work seems to play a subordinate role in predicting mental health does not mean that shift workers have the same level of well-being as other employees. Rather, in keeping with other findings (Tahghighi et al. 2017), negative effects of this type of work may depend on individual and job characteristics that we controlled for in our study (e.g., gender, education, or autonomy in occupational activity).

This study contributes to the developing body of research on the role of personal characteristics by underlining the direct impact and specifying the moderating role of personal resources in population-based data. Adding personal resources as predictors of mental health to the model slightly but significantly increased the explained variance. As argued above, the predictive value may seem weak at first glance. Nevertheless, self-esteem was a stronger predictor than each single job characteristic. Thus, our study provides further evidence for the prominent role of self-esteem as a predictor of well-being.

Contrary to our expectations, locus of control had no direct impact, a finding that can be interpreted in the light of the literature on bilocal expectancy as reviewed by Galvin et al. (2018): if external and internal loci of control are confounded in one measure, their effects can cancel each other out. Thus, the influence of control expectancies could have been underestimated in our study. Moreover, domain-specific approaches (e.g., work locus of control) should typically yield stronger relations with work-related factors than general locus of control (Wang et al. 2010).

In sum, including personal resources in the prediction of mental health did not alter the impact of most job characteristics, but there were some interactions as described below. The finding that the direct effect of autonomy in occupational activity disappeared when we controlled for personal resources may be due to reciprocal influences, but this is an issue that is beyond the scope of our study. It is possible that self-esteem is strengthened by this kind of occupational autonomy, which is based on vocational status, and vice versa.

The hypothesis that addressed the moderating effects of personal resources on job demands and job resources received partial support. The interaction between personal resources and job characteristics indicates that for people with medium or high levels of self-esteem, having the chance to be promoted was beneficial for their mental health. This finding underscores the idea that personal resources have a boosting effect (Bakker and Demerouti 2017): employees with good self-esteem feel more confident about their professional advancement and are more likely to grab opportunities for development, and this in turn can boost the positive effect of this job resource on their well-being. For very low levels of self-esteem, on the other hand, career opportunities are not beneficial, suggesting that they may be perceived as a threat or a goal that is impossible to attain and thus a source of frustration. In other words, in line with person-environment fit theory (Kristof-Brown et al. 2005), this finding suggests that employees function best when their personal resources match the characteristics of their jobs.

The second interaction reveals a buffering effect of personal resources with respect to job demands: Whereas mental health was impaired by working on weekends in respondents with low to medium levels of internal control, there were no such effects for people with high levels of control. The increasingly negative effect of lower internal control corresponds with Rotter’s (1966) notion that individuals feel more threatened by certain circumstances when they lack internal control.

However, the present study also shows that not all job characteristics are moderated by personal resources. Thus, first, there are some basic trends that are relevant to all employees. Second, in addition to increasing job resources and reducing job demands, it seems worthwhile to strengthen personal resources through training. Third, we should keep in mind that especially in the context of job resources such as good opportunities for promotion, one size does not fit all.

## Strengths and limitations

The strength of our study is its prospective design and the fact that we were able to draw on a large sample of German employees from companies of all sizes, branches, and regions. Further, we controlled for the baseline level of mental health and excluded participants who changed jobs during the study.

Nevertheless, there are several limitations. First, we used self-report measures for most predictors and the outcome, which means that the results are subject to potential biases. To alleviate this concern, we attempted to also include indicator-based variables. In fact, we observed consistent effects for all except one (internal locus of control) of the predictors assessed with the usual self-ratings, whereas only two (conflicts at work and working on weekends) of the six predictors that were based on factual information showed significant effects. This may either suggest that the effects of global self-ratings on self-rated mental health were overestimated due to common method bias or that the specific indicators in the present study, particularly the recall of supportive individuals and autonomy in occupational activity, were too narrow or focused on aspects that were less central for well-being than the global predictors. In any case, it may be worthwhile to continue examining detailed and indicator-based measures in future research in order to obtain more insights into specific starting points for preventive interventions such as team building or leadership training. To further address the possible weakness of self-reported data, different health outcomes (e.g., specific symptoms or mental health problems) should also be included in future studies. Furthermore, studying somatic health outcomes could help to reveal similarities and differences between effects on mental and somatic health. Differences in the relationships between job characteristics and health outcomes have previously been identified by Nixon et al. (2011) across physical symptoms, dimensions of burnout (Lee and Ashforth 1996), and well-being indicators (Limmer and Schütz 2018). Second, despite the use of longitudinal data and the adjustment for potential confounds, the possible impact of unmeasured third variables (e.g., coping strategies) or reciprocal relations (De Jonge et al. 2001) cannot be ruled out or specified. Still, if job characteristics have a causal effect on both personal resources and mental health, the observed partial correlations between job characteristics and mental health while controlling for personality may underestimate the true effects (Theorell et al. 2016). Further research is needed to more clearly differentiate between unidirectional, reciprocal, and reversed effects between personal resources, the work environment, and mental health. Shorter time intervals and a full panel design could help to clarify the causal relations. Furthermore, to reduce unmeasured biases, a latent factor analysis would be an improvement in future studies. In sum, more research is needed to confirm the causal connections behind our results and their relevance in the light of the small effect sizes.

## Practical implications

The present study suggests that worksite interventions involving personal resources may improve employee health in addition to the primary challenge of reducing health-impairing job demands. Our results highlight the overall relevance of time pressure, interruptions, and job insecurity in preventive efforts, including workplace risk assessment. Further exploration of the causality behind this association is important for specifying potential approaches in psycho-social risk assessment and avoidance. In addition, the findings on individual differences in the effects of job characteristics such as weekend work and the chance of being promoted can contribute to the drafting of interventions.

Apparently, one size does not fit all, and individual perceptions of demands as well as of well-intended incentives should be considered. For example, a choice between alternative incentives could be offered. Further, behavioral prevention may complement situational prevention because promoting personal resources in training and personnel development may help employees deal with the demanding aspects of their jobs and boost the effects of health-promoting job resources.

## Conclusion

This study investigated the power of ten job characteristics, two personal resources, and the interactions between jobs and individuals in predicting mental health in a large population-based sample. In line with previous empirical and theoretical reasoning, high time pressure, frequent interruptions, job insecurity, and conflicts predicted lower mental health. This implies that there are work characteristics that impair or enhance mental health across occupations regardless of other circumstances or personality. However, with other job characteristics such as opportunities for promotion or weekend work, the effects varied between individuals. This finding shows that not every demand is a burden for everyone, and not every incentive is beneficial for everyone. Such findings point to the importance of personal resources, which constitute a factor of personal resilience (Mäkikangas et al. 2004) such as self-esteem for health outcomes.

Even though there are limitations, the findings offer new insights into the advantages and difficulties involved in finding more objective measures of job characteristics. With respect to the practical implications, the results may offer a way to orient the assessment and development of healthy work designs.

## Electronic supplementary material

Below is the link to the electronic supplementary material.

Supplementary file1 (DOCX 44 kb)
