# Correction to: Interactive effects of personal resources and job characteristics on mental health: a population-based panel study

**DOI:** 10.1007/s00420-021-01690-2

**Published:** 2021-04-08

**Authors:** Anja Limmer, Astrid Schütz

**Affiliations:** 1 Department of Psychology, University of Bamberg, Bamberg, Germany; 2 Chair of Personality Psychology and Psychological Assessment, University of Bamberg, Markusplatz 3, 96047 Bamberg, Germany

## Correction to: International Archives of Occupational and Environmental Health 10.1007/s00420-020-01555-0

The article: Interactive effects of personal resources and job characteristics on mental health: a population-based panel study was written by Anja Limmer, Astrid Schütz was originally published electronically on the publisher’s internet portal (currently SpringerLink) on 6 June 2020 without open access.

With the author(s)’ decision to opt for Open Choice the copyright of the article changed on 15 February 2021 © The Author(s) 2021 and the article is forthwith distributed under the terms of the Creative Commons Attribution 4.0 International License (http://creativecommons.org/licenses/by/4.0/), which permits use, duplication, adaptation, distribution and reproduction in any medium or format, as long as you give appropriate credit to the original author(s) and the source, provide a link to the Creative Commons license and indicate if changes were made.

**Open Access** This article is distributed under the terms of the Creative Commons Attribution 4.0 International License (http://creativecommons.org/licenses/by/4.0/), which permits unrestricted use, distribution, and reproduction in any medium, provided you give appropriate credit to the original author(s) and the source, provide a link to the Creative Commons license, and indicate if changes were made.

The original article was corrected.

