# Supplementary material for: Interactive effects of personal resources and job characteristics on mental health: a population-based panel study
Source: Int Arch Occup Environ Health. 2020 Jun 6;94(1):43–53. doi: 10.1007/s00420-020-01555-0 (PMC8164588; doi:10.1007/s00420-020-01555-0)
Supplement: Supplementary file 1 — Supplementary file1 (DOCX 44 kb) [file 420_2020_1555_MOESM1_ESM.docx]

**Table S1**. Multiple regression results for predicting mental health in the full model (Step 4).

| *Predictors* |  | *b* | *p* | *SE* |
| --- | --- | --- | --- | --- |
| Baseline mental health |  | 0.44 | 0.000 | 0.017 |
| Age (years) |  | 0.03 | 0.033 | 0.012 |
| Male (0=no, 1=yes) |  | 0.53 | 0.046 | 0.266 |
| Education level |  |  |  |  |
| Upper secondary |  | 1.13 | 0.059 | 0.597 |
| Post-secondary |  | 0.83 | 0.220 | 0.677 |
| Bachelor or equivalent |  | 1.19 | 0.069 | 0.655 |
| Master/doctoral or equivalent |  | 0.79 | 0.288 | 0.739 |
| Leadership (0=no, 1=yes) |  | 0.34 | 0.231 | 0.281 |
| Working hours |  | -0.00 | 0.792 | 0.013 |
| Shift work (0=no, 1=yes) |  | 0.40 | 0.277 | 0.369 |
| Working on weekends (0=no, 1=yes) |  | -0.75 | 0.002 | 0.249 |
| Time pressure |  | -0.47 | 0.000 | 0.109 |
| Interruptions |  | -0.32 | 0.004 | 0.110 |
| Job insecurity |  | -0.33 | 0.032 | 0.154 |
| Conflicts at work (0=no, 1=yes) |  | -0.78 | 0.015 | 0.260 |
| Trust and career-support (0=no, 1=yes) |  | -0.18 | 0.489 | 0.321 |
| Autonomy in occupational activity |  | 0.27 | 0.087 | 0.155 |
| Chances of promotion |  | 0.35 | 0.002 | 0.113 |
| Self-esteem (SE) |  | 0.79 | 0.000 | 0.170 |
| Internal locus of control (ILoC) |  | -0.07 | 0.740 | 0.205 |
| SE x Working hours |  | 0.01 | 0.235 | 0.011 |
| SE x Shift work |  | 0.07 | 0.839 | 0.342 |
| SE x Working on weekends |  | -0.35 | 0.133 | 0.232 |
| SE x Time pressure |  | 0.15 | 0.121 | 0.098 |
| SE x Interruptions |  | -0.08 | 0.453 | 0.105 |
| SE x Job insecurity |  | -0.06 | 0.633 | 0.125 |
| SE x Conflicts at work |  | 0.52 | 0.072 | 0.288 |
| SE x Trust and career-support |  | 0.26 | 0.282 | 0.245 |
| SE x Autonomy |  | 0.09 | 0.458 | 0.121 |
| SE x Chances of promotion |  | 0.29 | 0.003 | 0.098 |
| ILoC x Working hours |  | -0.01 | 0.451 | 0.014 |
| ILoC x Shift work |  | 0.49 | 0.259 | 0.436 |
| ILoC x Working on weekends |  | 0.97 | 0.002 | 0.311 |
| ILoC x Time pressure |  | 0.04 | 0.791 | 0.135 |
| ILoC x Interruptions |  | 0.17 | 0.222 | 0.137 |
| ILoC x Job insecurity |  | 0.10 | 0.564 | 0.181 |
| ILoC x Conflicts at work |  | -0.45 | 0.266 | 0.404 |
| ILoC x Trust and career-support |  | 0.16 | 0.632 | 0.330 |
| ILoC x Autonomy |  | -0.02 | 0.879 | 0.156 |
| ILoC x Chances of promotion |  | -0.21 | 0.133 | 0.140 |
| Intercept |  | 48.82 | 0.000 | 0.636 |

*b* unstandardized coefficient, *SE* robust standard error.
